# Different Weight Loss Intervention Approaches Reveal a Lack of a Common Pattern of Gut Microbiota Changes

**DOI:** 10.3390/jpm11020109

**Published:** 2021-02-08

**Authors:** Carolina Gutiérrez-Repiso, María Molina-Vega, M. Rosa Bernal-López, Lourdes Garrido-Sánchez, José M. García-Almeida, Ignacio Sajoux, Isabel Moreno-Indias, Francisco J. Tinahones

**Affiliations:** 1 Unidad de Gestión Clínica de Endocrinología y Nutrición del Hospital Virgen de la Victoria, Instituto de Investigación Biomédica de Málaga (IBIMA), 29010 Málaga, Spain; gutierrezrepiso@gmail.com (C.G.-R.); molinavegamaria@gmail.com (M.M.-V.); lourgarrido@gmail.com (L.G.-S.); jgarciaalmeida@yahoo.com (J.M.G.-A.); fjtinahones@uma.es (F.J.T.); 2 Centro de Investigación Biomédica en Red de Fisiopatología de la Obesidad y la Nutrición (CIBERobn), Instituto de Salud Carlos III, 28029 Madrid, Spain; robelopajiju@yahoo.es; 3 Departamento de Medicina Interna del Hospital Regional Universitario de Málaga, Instituto de Investigación Biomédica de Málaga (IBIMA), 29009 Málaga, Spain; 4 Pronokal Group, Medical Department Pronokal, 08009 Barcelona, Spain; ignacio.s@pronokal.com; 5 Departamento de Medicina y Dermatología, Universidad de Málaga, 29010 Málaga, Spain

**Keywords:** gut microbiota, weight loss, Mediterranean diet, very-low-calorie ketogenic diet, bariatric surgery

## Abstract

Options for treatment of obesity include dietary approaches and bariatric surgery. Previous studies have shown that weight loss interventions have an impact on gut microbiota. However, a pattern of gut microbiota changes associated with weight loss independently of the type of intervention has not been described yet. This study includes 61 individuals who followed different weight loss strategies in three different trials: 21 followed a hypocaloric Mediterranean diet (MedDiet), 18 followed a very-low-calorie ketogenic diet (VLCKD) and 22 patients underwent sleeve gastrectomy bariatric surgery (BS). Gut microbiota profile was assessed by next-generation sequencing. A common taxon that had significantly changed within the three weight loss interventions could not be find. At the family level, *Clostiridiaceae* significantly increased its abundance with MedDiet and VLCKD, whilst *Porphyromonadacean* and *Rikenellaceae* significantly increased with VLCKD and BS. At genus level, in VLCKD and BS, *Parabacteroides* and *Alistipes* significantly increased their abundance whilst *Lactobacillus* decreased. At the species level, BS and VLCKD produced an increase in *Parabacteroides* *distasonis* and a decrease in *Eubactierium* *ventriosum* and *Lactobacillus* *rogosae*, whilst *Orodibacter* *splanchnicus* increased its abundance after the BS and MedDiet. Predicted metagenome analysis suggested that most of the changes after VLCKD were focused on pathways related to biosynthesis and degradation/utilization/assimilation, while BS seems to decrease most of the biosynthesis pathways. MedDiet was enriched in several pathways related to fermentation to short-chain fatty acids. Our results show that weight loss is not associated with a specific pattern of gut microbiota changes independently of the strategy used. Indeed, gut microbiota changes according to type of weight loss intervention.

## 1. Introduction

Relatively recently, we started to pay attention to the more than one hundred billion of microbial cells [1] that live inside and over us. The microbes that live in and on the human body are known as human microbiota and the genes contained in the microbiota constitute the microbiome. 

Obesity has become a worldwide health problem. Lifestyle changes, including a hypercaloric diet and a decrease in physical activity, are environmental key factors involved in the obesity epidemic. In the last years, microbiota has emerged as an important environmental factor that could contribute to obesity [2]. In this sense, fecal transplantation using animal models has arisen as a powerful research line to elucidate the role of gut microbiota in host metabolism [3].

Several studies have described the gut microbiota profile in obesity. Initial reports in animal models showed that obesity may be associated with a reduction in Bacteroidetes and an increase in Firmicutes [4]. The same tendency was observed in humans, although in a study made in twins showed in obese individuals a decrease in Bacteroidetes and an increase in Actinobacteria [5]. This assumption, initially accepted, is now considered incorrect, showing the bibliography contradictory results [6,7]. Proteobacteria phylum has been shown to be increased in obesity [8], although results are not conclusive, in several studies, an increase in members of this phylum as well as in the abundance of *Faecalibacterium, Osillospira, Akkermansia muciniphila y Faecalibacterium prausnitzii* has been reported [8]. Discrepancies between studies are mainly due to the different DNA extraction method, techniques used to analyze the gut microbiota and bioinformatic analysis pipelines.

Weight loss interventions induce changes in gut microbiota [9,10]. The first line of obesity treatment is by caloric restriction dietary approaches. Diet is one of the factors that most affect gut microbiota. In a recent review, Seganfredo et al. pointed out that similar dietary interventions such as hypocaloric, low carbohydrate or high protein diets, produced a reduction in *Roseburia* spp., *Eubacterium rectale* and other species belonging to the *Clostridium* Cluster XIVa, as well as a reduction in *Bifidobacterium* sp. On the contrary, the only trial consisting in non-hypocaloric but low-fat diet showed different results [11]. However, there is still an important gap in the research of different dietary interventions on gut microbiota.

Bariatric surgery is a powerful tool for weight loss achievement. Several studies have evaluated the relation between gut microbiota changes and the improvement in anthropometric and metabolic variables after bariatric surgery. Animal and human studies have reported common changes after bariatric surgery such as a decrease in the abundance of Firmicutes and an increase in Bacteroidetes, Proteobacteria and its class *Gammaproteobacteria* (order *Enterobacteriales*, family *Enterobacteriaceae*, genus *Escherichia*) [12]. Also, gut microbiota is affected by bariatric surgery in a procedure manner, higher levels of *Akkermansia*, *Eubacterium*, *Hemophilus* and *Blautia* have been shown in sleeve gastrectomy, whilst in Roux-en-Y gastric bypass *Veillonella*, *Slackia*, *Granucatiella* and *Acidaminococcus* occurred with greater levels [13].

Based on the previous studies, it is difficult to establish a pattern of gut microbiota changes associated with weight loss independently of the type of intervention, if there is such a pattern. The aim of the present study is identifying similar patterns in gut microbiota changes related to weight loss using three completely different strategies.

## 2. Results

Table 1 contains detailed anthropometric and biochemical characteristics of the groups. Age was significantly different in the three groups: 64.0 ± 4.7 years in Mediterranean diet (MedDiet), 47.5 ± 5.5 years in bariatric surgery (BS) and 42.6 ± 10.8 years in the very-low-calorie ketogenic diet (VLCKD) group (*p* < 0.001). At baseline, as it was expected, weight, BMI, waist circumference and C-reactive protein levels (CRP) were statistically higher in BS group than in the other groups.

The three interventions produced a statistically significant decrease in weight, body–mass index (BMI), waist circumference and triglyceride levels (*p* < 0.001 in all cases, except triglycerides *p* = 0.001), although the reduction was higher in BS, followed by VLCKD and MedDiet.

BS intervention produced a statistically significant decrease in glucose levels (*p* < 0.001), HbA1c (*p* = 0.001), systolic blood pressure (*p* = 0.021) and CRP levels (*p* = 0.008). MedDiet produced a statistically significant decrease in cholesterol (*p* = 0.016), high density lipoprotein cholesterol (HDL-cholesterol)(*p* = 0.023), low density lipoprotein cholesterol (LDL-cholesterol) (*p* = 0.018) and HbA1c levels (*p* < 0.001), whilst VLCKD produced a statistically significant decrease in cholesterol (*p* = 0.026), systolic and diastolic blood pressure (*p* = 0.001 and *p* = 0.020, respectively); while ketone bodies and zonulin levels increased significantly (*p* = 0.011 and *p* = 0.027, respectively).

### 2.1. Changes in Gut Microbiota Diversity after Weight Loss Interventions

β-diversity was qualitatively and quantitatively evaluated using the unweighted and weighted UniFrac distances, respectively, and was visualized as principal coordinate analysis plots in order to visualize complex relationships. None of the interventions produced statistically different changes in β-diversity indexes (data not shown).

**Figure 1:**
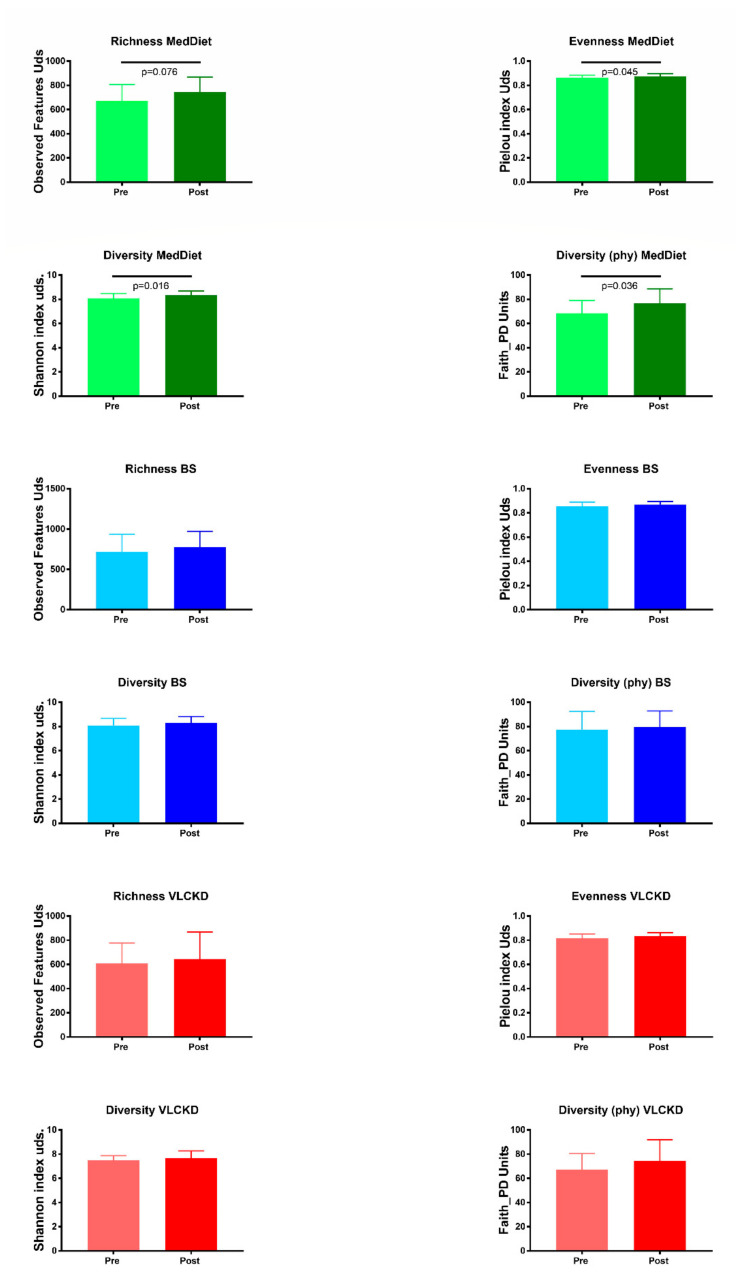
α-diversity indexes at baseline and the end of the study in the three interventions. MedDiet: Mediterranean Diet group (*n* = 21). BS: Bariatric surgery group (*n* = 22). VLCKD: very-low calorie ketogenic diet group (*n* = 18). Pre: at baseline. Post: at the end of the intervention.

However, the MedDiet intervention produced an increase in α-diversity. Evenness, calculated by the Pielou index (*p* = 0.045) and biodiversity, estimated by the Shannon (*p* = 0.016) and Faith_pd index (*p* = 0.036), increased significantly with the intervention. No significant differences were found with the other interventions (Figure 1).

### 2.2. Changes in Gut Microbiota Profile after Weight Loss Interventions

Bugbase algorithm-base prediction is able to classify 16S rRNA sequences by their characteristics in groups. Bugbase suggested that during weight loss bacteria were differently affected according to each procedure: form biofilms taxa decreased in VLCKD group (*p* = 0.049), whilst in MedDiet group, a decrease in potentially pathogenic and stress tolerant taxa was suggested (*p* = 0.027 and *p* = 0.013, respectively) (Table 2).

**Figure 2:**
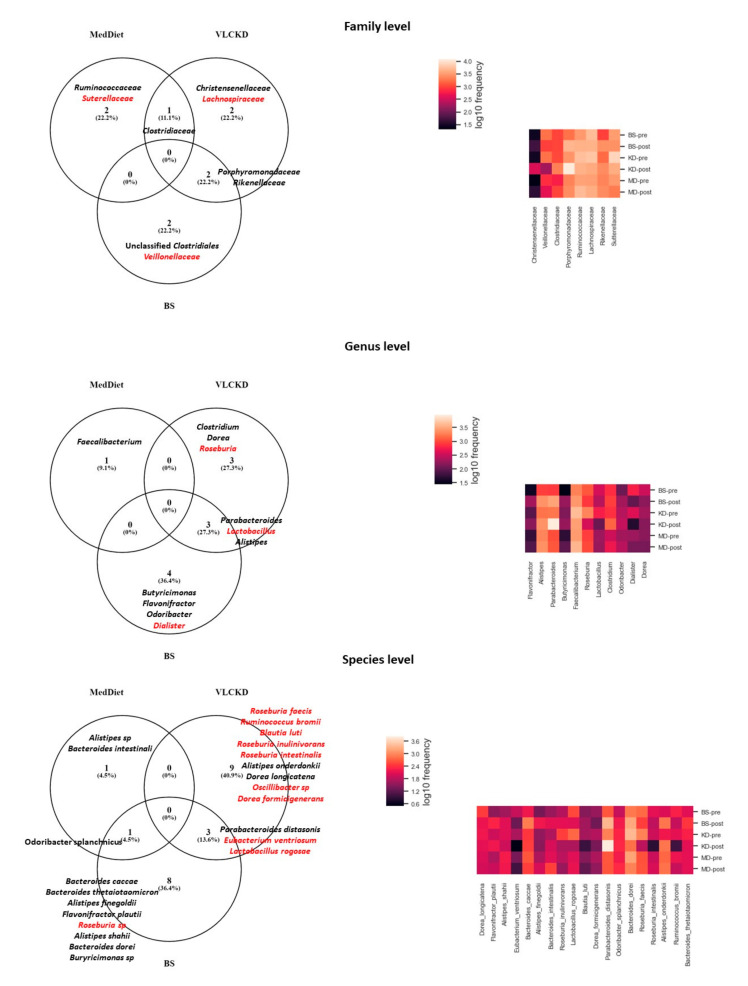
Taxon that changed significantly in each intervention at different taxonomic levels. In red color: taxon that decreased their abundance at the end of the intervention. In black color: taxon that increased their abundance at the end of the intervention. MedDiet: Mediterranean Diet group (*n* = 21). BS: Bariatric surgery group (*n* = 22). VLCKD: very-low calorie ketogenic diet group (*n* = 18).

Going through the particular taxa changes, in Figure 2 are represented the significant changes (LEfSe analysis, LDA > 3, *p* < 0.05) at different taxonomic level after each intervention. Although some changes were particular of a specific intervention, the most striking result was that we could not identify a common taxon that had significantly changed within the three weight loss interventions.

However, some interventions shared particular changes, although never among the three treatments. At the family level, *Clostiridiaceae* significantly increased its abundance with MedDiet and VLCKD, whilst *Porphyromonadacean* and *Rikenellaceae* significantly increased with VLCKD and BS. At genus level, in VLCKD and BS, *Parabacteroides* and *Alistipes* significantly increased their abundance whilst *Lactobacillus* decreased. At species level, BS and VLCKD produced an increase in *Parabacteroides distasonis* and a decrease in *Eubactierium ventriosum* and *Lactobacillus rogosae*, whilst *Orodibacter splanchnicus* increased its abundance after BS and MedDiet (Figure 2).

### 2.3. Common Core Microbiome at the End of the Weight Loss Interventions

**Figure 3:**
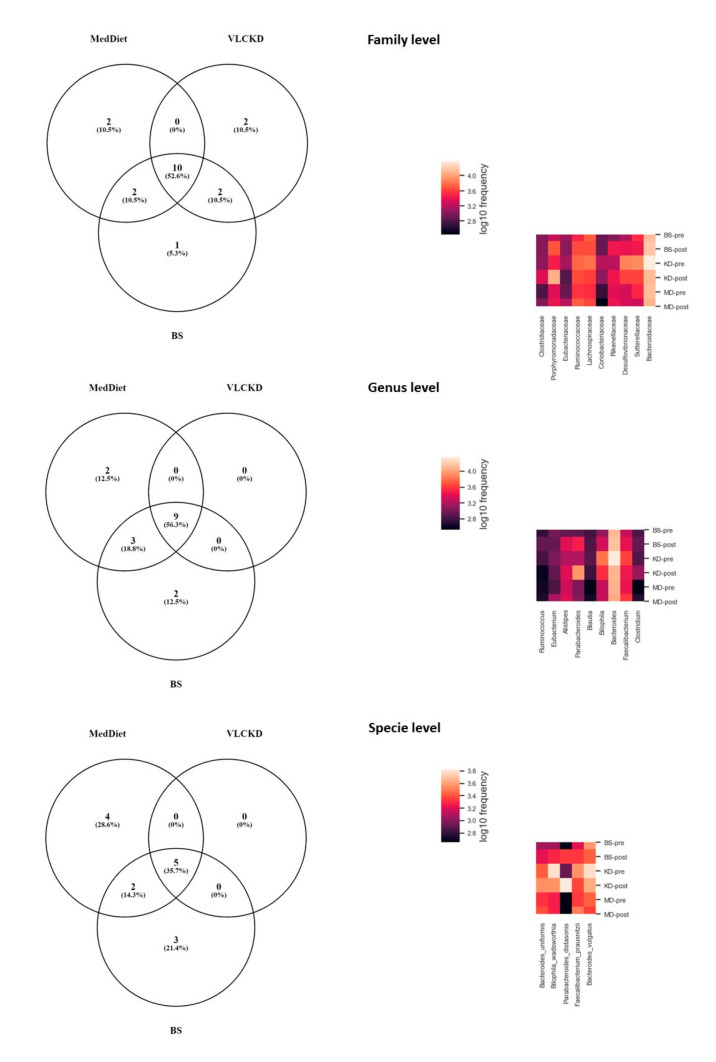
Venn diagram of the core microbiomes shared among the 85% of the samples at different taxonomic level. MD-pre: Mediterranean Diet at baseline. MD-post: Mediterranean Diet at the end of the intervention (*n* = 21). BS-pre: Bariatric surgery at baseline. BS-post: Bariatric surgery at the end of the intervention (*n* = 22). VLCKD-pre: very-low calorie ketogenic dietat baseline. VLCK-post: very-low calorie ketogenic diet at the end of the intervention (*n* = 18).

After the lack of any particular taxon characteristic of weight loss and shared by the three procedures, we wondered if each intervention provoked changes through a concrete core gut microbiome profile. For this purpose, we examined the core microbiome of each group, meaning those features that were shared among the 85% of the samples of each study group after the intervention. and investigated if some of these core taxa were shared among the interventions. At the family level, we found a common core microbiome shared by the three interventions that comprises 10 families: *Bacteroidaceae, Ruminococcaceae, Lachnospiraceae, Porphyromonadaceae, Desulfovibrionaceae, Sutterellaceae, Rikenellaceae, Eubacteriaceae, Clostridiaceae and Coriobacteriaceae* (Figure 3). Only *Rikenellaceae* and *Clostridiaceae* increased significantly in two weight loss interventions (*Rikenellaceae* in VLCKD and BS; *Clostridiaceae* in MedDiet and VLCKD) (Figure 2). When we checked how this core microbiome changed within each weight loss intervention, we found statistically significant differences between the three weight loss methods. *Bacteroidaceae* family decreased its abundance in VLCKD compared to BS procedure (*p* = 0.031), whilst *Clostridiacea* increased significantly in VLCKD compared to BS (*p* = 0.049). *Lachnospiraceae* significantly increased its abundance in MedDiet compared to VLCKD (*p* = 0.029). *Porphyromonadaceae* abundance was higher in VLCKD than in MedDiet (*p* < 0.001) and BS (*p* = 0.003) and it was higher in BS than in MedDiet (*p* = 0.002) (Supplementary Figure S1a).

At genus level, the common core microbiome after the interventions comprised 9 genera: Bacteroides, Faecalibacterium, Bilophila, Alistipes, Parabacteroides, Eubacterium, Ruminococcus, Blautia and Clostridium (Figure 3). Only Alistipes and Parabacteroides increased in two interventions (BS and VLCKD) (Figure 2). When we analyzed the changes in this core microbiome through the interventions, we found that Bacteroides decreased its abundance in VLCKD compare to BS (*p* = 0.023), whilst Ruminococcus decreased in VLCKD compared to MedDiet (*p* = 0.020). Parabacteroides increase was significantly higher in VLCKD compare to BS (*p* = 0.011) and MedDiet (*p* < 0.001), this increase was also higher in BS compared to MedDiet (*p* = 0.001) (Supplementary Figure S1b).

At species level, the common core microbiome after the interventions comprised 5 species: Faecalibacterium prausnitzii Bilophila wadsworthia, Bacteroides vulgatus, Bacteroides uniformis and Parabacteroides distasonis. Only P. distasonis increased its abundance in two interventions (BS and VLCKD) (Figure 2). When we analyzed the changes of P. distasonis through the interventions, we found that its increase was significantly higher in VLCKD compare to BS (*p* = 0.003) and MedDiet (*p* < 0.001) and also was higher in BS compared to MedDiet (*p* = 0.003) (Supplementary Figure S1c).

### 2.4. Functional Analysis of Predicted Metagenomes after Weight Loss Interventions

**Figure 4:**
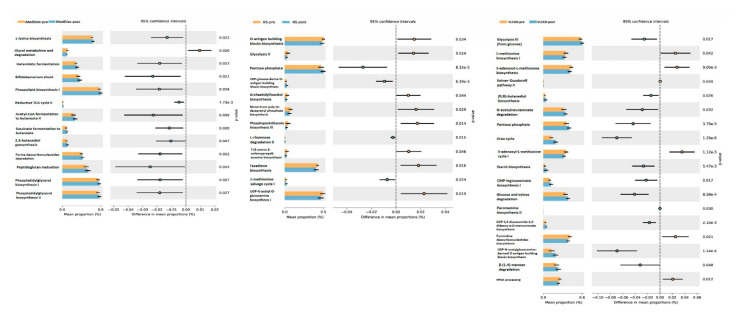
Relative abundance of the Kyoto Encyclopedia of Genes and Genomes (KEGG) pathways that changed significantly after each intervention. MD-pre: Mediterranean Diet at baseline. MD-post: Mediterranean Diet at the end of the intervention (*n* = 21). BS-pre: Bariatric surgery at baseline. BS-post: Bariatric surgery at the end of the intervention (*n* = 22). VLCKD-pre: very-low calorie ketogenic diet at baseline. VLCK-post: very-low calorie ketogenic diet at the end of the intervention (*n* = 18).

One step forward in the understanding of gut microbiota changes in weight loss is to know what this gut microbiota is doing as a metabolic organ. We inferred these metabolic pathways with a Metacyc pathway analysis with PiCRUSt2. Although Metacyc pathway did not show significative changes in common pathways to the three interventions, particular trends were observed in each intervention, represented in Figure 4. VLCKD was the intervention that produced more changes. In general, pathways related to biosynthesis and degradation/utilization/assimilation seemed to be increased with the VLCKD. Several pathways involved in sugar biosynthesis were enriched whilst pathways involved in L-methionine biosynthesis decreased. MedDiet was characterized by the enrichment in pathways relative to biosynthesis and generation of precursor metabolites and energy and more particularly related to fermentation, namely heterolactic fermentation, Bifidobacterium shunt, acetyl-CoA fermentation to butanoate II and succinate fermentation to butanoate. Finally, BS seemed to be characterized by a decrease in pathways relative to biosynthesis. 

Some trends were shared by interventions. In VLCKD and BS, a decrease in nucleic acid processing pathway was suggested, whilst sugar biosynthesis and pentose phosphate pathways were shown to be enriched. Butanediol biosynthesis pathway was enriched in both MedDiet and VLCKD. In Supplementary Table S1 are detailed all the pathways altered in each intervention.

## 3. Discussion

Gut microbiota has emerged as a complex organ in which interrelationships among its members and the host are environmentally influenced. Our study shows that weight loss was not associated with a particular pattern of gut microbiota changes independently of the strategy used. Indeed, gut microbiota changed according to the weight loss intervention.

In a population level, diversity analyses have shown that there were not homogeneous changes in gut microbiota diversity after weight loss intervention since gut microbiota diversity increased only with MedDiet. There is not consensus about the effect of weight loss intervention on gut microbiota diversity, as it has been shown by the contradictory results. Previous studies reported that weight loss interventions increased gut microbiota diversity although others showed no impact [11].

Although we could not find common changes in gut microbiota profile in the three interventions, we could describe significant changes shared pairwise. VLCKD and BS presented some similarities. At family level, *Porphyromonadaceae* and *Rikenellaceae* increased with both interventions, also in our results, *Rikenellaceae* was one of the members of the core microbiome after the three interventions. Both bacteria have been negatively correlated with body mass index and fat mass and their abundance has been reported to be increased after bariatric surgery [14]. Also, in animal models, these bacteria have been associated with weight loss using probiotics [15].

At genus level, our study showed that *Parabacteroides*, member of *Porphyromonadaceae* family and *Alistipes*, member of *Rikenellaceae* family, increased after VLCKD and BS, while *Lactobacillus* decreased. *Parabacteroides* and *Alistipes* were represented in the common core microbiome after the three interventions. Previous studies showed that were negatively associated with waistline and body mass index in adults [14,16] and young people [17]. While an increase of *Alistipes* at baseline was shown to be a predictor of successful weight loss after very-low-calorie interventions [18].

In contrast, *Lactobacillus* belongs to Firmicutes phylum and its abundance has been shown to be increased in patients with obesity [11]. Also, many studies have reported a decrease in *Lactobacillus* abundance after bariatric surgery [19]. However, despite this association, it appears that some of the bacteria of this phylum have a protective effect against weight gain, especially through its probiotic action [20]. 

At species level, after VLCKD and BS, *Parabacteroides distasonis* increased its abundance while *Eubacterium ventriosum* and *Lactobacillus rogosae* decreased. *P. distasonis* was presented in the common core microbiome after the interventions. Previous studies showed a negative correlation between *P. distasonis* obesity and metabolic syndrome [21]. *P. distasonis* has been suggested to have metabolic benefits on decreasing weight gain, hyperglycemia and hepatic steatosis in ob/ob and high-fat diet (HFD)-fed mice [22]. *P. distasonis* alleviates metabolic dysfunctions via production of succinate and secondary bile acids [22]. Also, after long-term consumption of the Mediterranean Diet its abundance has been shown to be increased [10]. Few studies evaluate the possible association of *Eubacterium ventriosum* and *Lactobacillus rogosae* with obesity or weight loss, except for previous reports that have associated *Eubacterium ventriosum* with obesity [23,24].

Changes in the gut microbiota profile produced after MedDiet were more specific and less similar to the changes observed with the other interventions. The common features were an increase in *Clostridicaceae* in both VLCKD and MedDiet and an increase in *Odoribacter splanchnicus* in both BS and MedDiet. *Clostridiaceae* was also a member of the common gut microbiota described after the three interventions. *Clostridiaceae* belongs to the phylum Firmicutes, which has been traditionally reported to be decreased in obesity [25], although *Clostridiaceae* has been also reported to decrease after bariatric surgery [26] and changes in the abundance of genera and species belonging to this family have been associated to weight loss changes [11]. So far, there is little information about the relation between *Odoribacter splanchnicus*, obesity and weight loss; a previous study showed that its abundance increased after Roux-en-Y gastric bypass [27].

In the absence of shotgun metagenomic sequencing data, we applied PICRUSt to our 16S rRNA gene analysis to predict metagenome functional content. PICRUSt is a computational approach that uses evolutionary modelling to predict the present gene families from 16S data and a reference genome database. According to the PICRUSt analysis, few studies have evaluated the effect of weight loss on functional pathways of the gut microbiota. Our results did not show a significant change in functional gut microbiota pathways common to the three interventions. This fact could indicate that gut microbiota adapted to the new environmental conditions of caloric restriction and nutrients specific of each intervention. In general, VLCKD showed most of the changes focused on pathways related to biosynthesis and degradation/utilization/assimilation what could indicate a change in their metabolism, while BS seems to decrease most of the biosynthesis pathways, a possible sign of the extremely caloric restriction. However, similar to taxonomic results, some pathways were pairwise affected by these two interventions. Pentose phosphate and sugar biosynthesis pathways, both related to carbohydrate metabolism, were enriched after VLCKD and BS, while a decrease in nucleic acid processing pathway was suggested. Pentose phosphate pathway has recently been suggested to be associated with obesity, although there are contradictory results: in infants born of normo-weight mothers, it was suggested to be increased [28] but also in animal models of obesity [29]. 

On the other hand, nucleic acid processing involves tRNA processing, tRNAs are essentials for protein synthesis, Recent evidence suggest that bacterial microorganisms can release extracellular vesicles made up of tRNAs and other RNAs. These extracellular vesicles have been implicated in the induction of inflammatory responses and bacterial pathogenesis, among other functions [30]. 

But VLCKD and MedDiet also shared some pathways. Butanediol biosynthesis pathway was enriched in both VLCKD and MedDiet. Butanediol is an organic compound and a primary alcohol. Its metabolic function is not fully understood, although it has been suggested that it may play a role in preventing intracellular acidification by changing the metabolism from acid production to the formation of a neutral compound [31]. 

In animal models, it has been shown that ketone ester *R*,*S*-1,3-butanediol with diacetoacetate increases circulating ketone concentrations, contributing to reduce body weight and adiposity [32] and also increase resting energy expenditure and markers of brown and white adipose thermogenesis in lean mice [33]. Moreover, butanediol is converted to D-β-hydroxybutirate, by liver aldehyde and alcohol dehydrogenases, which has been suggested to decrease plasma ghrelin levels and, also to be a direct mediator of appetite suppression [34].

Though the aim of the present study was not highlighting the changes induced by MedDiet compared the changes produced by the other interventions, we found that MedDiet was enriched in several pathways related to fermentation to short-chain fatty acids (SCFAs). SCFAs are produced by microbial fermentation of undigested carbohydrates. host, environmental, dietary and gut microbiota factors influence on the amount of SCFAs produced [35]. There are contradictory results about the relationship between obesity and SCFAs. However, a recent meta-analysis concluded that obesity was associated with high levels of SCFAs [36]. Several studies have shown that weigh loss induced by low carbohydrate diet and bariatric surgery, may lead to a decrease in SCFAs [37,38]. Nevertheless, high adherence to Mediterranean Diet has also been associated with increased SCFAs concentrations [39]. There are evidence showing the beneficial effect of SCFAs on cardiometabolic health. In an animal study, SCFAs (especially butyrate) were shown to prevent the translocation of LPS, a potent inflammatory molecule produced in the cell membrane of gram-negative bacteria [40]. In overweight patients, it has been shown the involvement of SCFAs in appetite regulation, the administration of SCFAs, inulin-propionate, increased postprandial plasma PYY and glucagon-like peptide-1, significantly reducing weight gain and adiposity [41]. 

Our study has several limitations. This is not a randomized clinical trial but it is difficult to randomize patients undergoing bariatric surgery. At baseline the populations are different, as we expected, patients undergoing bariatric surgery have an extreme phenotype. Also MedDiet group was significantly older and age may have an impact on gut microbiota. However, the aim of the study was to analyze different populations undergoing different interventions, trying to elucidate a common gut microbiota profile associated with weight loss and independent of the population and the type of intervention. Another limitation is the duration of the follow-up that varies between groups because of differences in the procedures used for weight loss. Both VLCKD and BS got a significant weight loss in a shorter period of time, however Mediterranean intervention used in this study required a wider period of time in order to get a relevant weight loss. The strength of the study is its pioneering approach trying to find a weight loss patterns with volunteers that belong to the same region with similar sociodemographic characteristics, as well as all the samples were analyzed using the same protocol and results were also analyzed using the same bioinformatic pipelines.

In conclusion, our results showed that there were not common changes in gut microbiota profile associated with weight loss. The analysis of predicted metagenome confirmed this assumption, observing the affection of different pathways in each intervention. Changes in gut microbiota profiles and their functionality depended on the type of weight loss intervention. In the last years, the possibility that gut microbiota manipulation may be used in achieving sustained weight loss has been launching gut microbiota studies in the obesity field. However, results from this study suggest that this manipulation, although possible, should be formulated in accordance to the intervention followed, supporting the role of gut microbiota in precision medicine.

## 4. Material and Methods

This study includes 61 individuals who followed different weight loss strategies in three different trials: 21 followed a hypocaloric Mediterranean diet, 18 followed a very low-calorie ketogenic diet (VLCKD) and 22 patients underwent sleeve gastrectomy bariatric surgery. The sample size was established based on previous published articles from the group [9,13] where changes in gut microbiota produced by VLCKD and BS were evaluated, using a sample size in the groups of 9 and 14, respectively. So, we decided to include at least 14 participants per group in order to guarantee the statistical power. Exclusion criteria in the three studies were the use of antibiotics, probiotic or prebiotic agent which could modify microbiota in the previous three months, aged under 18 and over 75, patients with cardiovascular, neurodegenerative disease, acute inflammatory, infectious disease or known type 2 diabetes mellitus. The studies were conducted in accordance with the Declaration of Helsinki, all protocols were approved by the Biomedical Research Ethic Coordinator Committee of Andalucía (CCEIBA) and all participants provided written informed consent. All interventions were followed by a physician and a dietician and patients kept food records that helped to assess their diet and make recommendations. The details of each intervention are described as follows:

*Hypocaloric diet* (MedDiet): The participants were recruited from 2013 to 2016 at the Endocrinology and Nutrition Department of the Virgen de la Victoria University Hospital (Málaga, Spain). The participants were enrolled in a lifestyle weight-loss intervention program consisting on a hypocaloric Mediterranean diet and a recommendation of physical activity for six months. The hypocaloric diet was based on a reduction of about 600 kcal in the energy intake with a calorie distribution as follows: 35–40% fats (8–10% saturated fatty acids), 40–45% carbohydrates and 20% protein. Additionally, daily exercise practicing was recommended to all participants, which involved walking on average for 150 min every week throughout the study. The dietary and physical intervention involved individual appointments with a nutritionist every week during the first two months, followed by monthly visits during the next four months [42]. Stool samples were obtained prior intervention and six months after. 

*Very low-calorie ketogenic diet* (VLCKD): The participants were recruited from August 2016 to November 2016 at the Endocrinology and Nutrition Department of the Virgen de la Victoria University Hospital (Málaga, Spain). All participants followed a VLCKD according to a commercial weight-loss program (PnK method by Pronokal Group; http://www.pronokalgroup.com), which includes lifestyle and behavioral modification support [9]. All intervention was supervised by a specialist physician and assessed by an expert dietician and the protocol ensures the appropriate amount of proteins to prevent the loss of lean mass. VLCKD (600–800 kcal per day) is low in carbohydrates and lipids. This method is based on high biological-value protein preparations that contain 15 g protein, 4 g carbohydrates, 3 g fat and 50 mg docosahexaenoic acid and provide 90–110 kcal. The VLCKD is divided into three phases and supplements consisting of vitamin and mineral supplements, such as K, Na, Mg, Ca and omega-3 fatty acids, were provided during the intervention. In first phase of the VLCKD, patients consumed high-biological-value protein preparations five times a day together with low glycemic index vegetables. Thus, in the second stage, one of the protein preparations was substituted with a natural protein (e.g., meat or fish) either at lunch or at dinner. In the third phase, a second protein preparation was replaced by a serving of a low-fat natural protein. VLCKD was maintained for 2 months and stool samples were obtained prior intervention and two months after. 

Participants received no monetary incentive. This trial was registered at www.clinicaltrials.gov as NCT03530501.

*Bariatric surgery* (BS): The study was performed in patients with morbid obesity who underwent sleeve gastrectomy bariatric surgery between May 2015 and March 2017 at the Virgen de la Victoria University Hospital (Málaga, Spain) [13]. After surgery, patients received recommendations about diet, patients started with a liquid diet for 1–2 weeks, followed by crushed or semi-soft diet for 2 weeks. In the next following weeks post-surgery, solid diet was introduced progressively. During all these weeks, patients received protein supplementation to prevent protein malnutrition Stool samples were obtained prior intervention and three months after the surgery.

### 4.1. Anthropometric and Laboratory Measurements

Weight and height were measured according to standardized procedures and body mass index (BMI) was calculated as weight (kg)/height^2^ (m^2^).

At different study points, blood samples were collected after a 10–12 h fast. The serum was separated and immediately frozen at −80 °C until analysis. Serum biochemical parameters were measured in duplicate using enzymatic methods.

### 4.2. Gut Microbiota Analysis

Hands on microbiome analyses are explained in detail in the Supplementary Material and Methods. In brief, DNA was extracted from feces and libraries from the 16S rRNA gene were built with the 16S Metagenomics kit, posteriorly templated on the automated Ion Chef system followed by sequencing on an Ion S5 (ThermoFisher Scientific, Waltham, MA, USA). 

### 4.3. Sequence Data and Statistical Analysis

Raw 16S rRNA sequencing data for all samples have been deposited in the NCBI short read archive under ID study: PRJNA634244. Quality sequences were further translated into amplicon sequence variants (ASVs) using DADA2 with adapted parameters for Ion Torrent data [43] within the microbiome analysis package QIIME2 (www.qiime2.org) [44], which will also be used for diversity analysis with the diversity plugin. α-diversity (intra-community diversity) was measured using richness (Shannon, Faith_pd and observed ASVs) and evenness (Pielou) indexes β-diversity (inter-communities diversity) was measured using Unweighted UniFrac distance (qualitative measure) and Weighted UniFrac distance (quantitative measure). Taxonomic analysis was assessed through the 16S rRNA Profiling within the tool Ion Reporter (Ion Reporter Software 5.12, ThermoFisher) clustering with the reference base Greengenes version 13_5 at 99% of identity and the curated MicroSEQ^®®^ 16S Reference Library V2013.1. OTU-tables at the different taxa levels were introduced within the webtool MicrobiomeAnalyst [45], where the data filtering and normalization steps were performed. Differential abundance analysis will be assessed with LEFSe within MicrobiomeAnalyst with the default parameters of the developer [46]. These OTU-tables were further analyzed with QIIME2 to calculate the core-microbiomes of each intervention with the plugin feature-table. Further visualization was performed with the web tool Venny [47]. Phylogenetic Investigation of Communities by Reconstruction of Unobserved States plugin (PICRUSt2) was used to predict metagenome function within QIIME2. MetaCyc pathways [48] were normalized within QIIME2 and further analyzed with STAMP [49]. A further analysis of the Organism-level microbiome phenotype prediction was obtained by BugBase [50].

Statistical software package SPSS version 22.0 (SPSS Inc., Chicago, IL, USA) was used to study differences in anthropometric and biochemical variables. Differences between the groups were analyzed by a one-way ANOVA followed by a Duncan’s post hoc test and Wilcoxon signed-rank test was used to calculate differences between baseline and the end of the intervention. Values were considered statistically significant when *p* or *q* value < 0.05.

## Figures and Tables

**Table 1:** Anthropometric and biochemical variables.

	Mediterranean Diet *n* = 21	Bariatric Surgery *n* = 22	VLCKD *n* = 18
Sex (M/F)	10/11	9/13	8/10
Age (years)	64.0 (4.7) ^a^	47.5 (5.5) ^b^	42.6 (10.8) ^c^
Weight (kg)			
Pre	88.1 (11.2) ^a^	128.5 (17.4) ^b^	93.1 (10.2) ^a^
Post	80.3 (10.9) ***	104.6 (13.2) ***	80.2 (7.4) ***
Change	−7.8 (1.9) ^a^	−23.9 (7.5) ^b^	−12.9 (3.0) ^c^
BMI (kg/m^2^)			
Pre	33.4 (3.3) ^a^	45.0 (5.0) ^b^	33.0 (1.4) ^a^
Post	30.6 (3.3) ***	37.3 (4.3) ***	28.5 (1.3) ***
Change	−2.7 (0.9) ^a^	−7.9 (2.0) ^b^	−4.5 (0.7) ^c^
Waist circumference (cm)			
Pre	112.0 (8.1) ^a^	131.7 (11.1) ^b^	110.4 (6.5) ^a^
Post	104.2 (8.1) ***	116.9 (9.2) ***	97.8 (6.6) ***
Change	−7.7 (2.5) ^a^	−15.9 (5.6) ^b^	−12.5 (4.4) ^c^
Glucose (mg/dL)			
Pre	106.2 (18.9) ^a^	112.7 (33.8) ^a^	87.2 (9.4) ^b^
Post	100.3 (10.3)	88.4 (12.6) ***	83.1 (9.2)
Change	−5.9 (17.4) ^a^	−24.0 (27.5) ^b^	−4.0 (10.8) ^a^
Total cholesterol (mg/dL)			
Pre	213.3 (32.6) ^a^	191.4 (22.4) ^b^	203.9 (31.6) ^ab^
Post	198.2 (35.6) *	186.3 (23.6)	185.8 (30.0) *
Change	−10.7 (25.9)	−3.3 (20.7)	−18.0 (29.3)
Triglycerides (mg/dL)			
Pre	155.1 (45.2)	206.2 (181.4)	137.1 (63.2)
Post	119.7 (46.3) **	123 (38.7) **	83.1 (23.9) **
Change	−32.7 (37.4)	−85.4 (170.5)	−54.0 (59.2)
HDL-chol (mg/dL)			
Pre	51.3 (12.7)	45.6 (10.1)	53.5 (13.1)
Post	56.2 (13.2) *	45.9 (9.8)	53.2 (12.7)
Change	5.3 (9.3)	0.1 (8.5)	0.7 (7.4)
LDL-chol (mg/dL)			
Pre	131.1 (31.6)	109.5 (23.4)	125.4 (30.5)
Post	118.0 (33.6) *	115.8 (21.5)	116.0 (27.3)
Change	−9.7 (21.8) ^a^	8.4 (23.4) ^b^	−7.2 (24.4) ^a^
HbA1c (%)			
Pre	5.8 (0.5) ^a^	5.9 (0.9) ^a^	5.3 (0.2) ^b^
Post	5.5 (0.2) ***	5.3 (0.3) **	5.1 (0.2)
Change	−0.3 (0.3)	−0.5 (0.8)	−0.3 (0.2)
SBP (mm Hg)			
Pre	138.2 (12.4)	140 (22.2)	129.4 (16.5)
Post	132.1 (13.0)	132.4 (21.7) *	118.3 (11.9) **
Change	−6.0 (13.5)	−6.7 (20.9)	−11.0 (10.8)
DBP (mm Hg)			
Pre	75.7 (10.2) ^a^	85.8 (10.8) ^b^	79.5 (7.4) ^a^
Post	73.7 (10.8)	82.1 (12.9)	74.3 (8.7) *
Change	−2.0 (7.9)	−4.2 (14.3)	−5.1 (7.7)
Ketone bodies (mmol/L)			
Pre	0.20 (0.03)	0.31 (0.14)	0.42 (0.72)
Post	0.25 (0.15)	0.31 (0.25)	0.67 (1.09) *
Change	0.04 (0.14) ^a^	0.006 (0.25) ^a^	0.24 (0.45) ^b^
CRP (mg/dL)			
Pre	1.63 (1.54) ^a^	6.61 (4.16) ^b^	3.80 (3.64) ^c^
Post	1.63 (1.60)	4.89 (3.93) **	3.19 (2.57)
Change	−0.004 (1.57) ^a^	−1.72 (3.56) ^b^	−0.12 (1.65) ^ab^
Zonulin (ng/mL)			
Pre	259.3 (63.4)	274.8 (42.1)	302.1 (144.1)
Post	243.3 (86.8)	295.6 (59.5)	379.4 (188.5) *
Change	−16.0 (70.4) ^a^	20.8 (56.5) ^ab^	66.9 (120.5) ^b^

Results as given as mean (standard deviation). BMI: Body mass index. SBP: systolic blood pressure. DBP: diastolic blood pressure. CRP: C reactive protein. Differences between the groups were analyzed by a one-way ANOVA followed by a Duncan’s post hoc test. Values with different uppercase letters (^a,b,c^) indicate statistically significant difference between the groups (*p* < 0.05) at Duncan’s test. Wilcoxon signed-rank test was used to calculate differences between baseline and the end of the intervention: *** *p* < 0.001, ** *p* < 0.01, * *p* < 0.05.

**Table 2:** Organism-level microbiome phenotype prediction.

	Mediterranean Diet *n* = 21	Bariatric Surgery *n* = 22	VLCKD *n* = 18
Aerobic			
Pre	0.0668 (0.04514)	0.0621 (0.0480)	0.0932 (0.0673)
Post	0.0453 (0.0334)	0.0499 (0.0362)	0.0668 (0.0509)
Change	−0.0215 (0.0413)	−0.0144 (0.0571)	−0.0237 (0.0631)
Anaerobic			
Pre	0.8007 (0.1198)	0.8294 (0.1133)	0.7127 (0.1307)
Post	0.8074 (0.1017)	0.7968 (0.1348)	0.7505 (0.1107)
Change	0.0067 (0.1089)	−0.0269 (0.1642)	0.0406 (0.1448)
Contains Mobile Elements			
Pre	0.1518 (0.0865)	0.1291 (0.0611)	0.2026 (0.1305)
Post	0.1152 (0.0486)	0.2012 (0.1746)	0.2427 (0.1094)
Change	−0.0365 (0.0842) ^a^	0.0672 (0.1658) ^b^	0.0402 (0.1117) ^ab^
Facultatively Anaerobic			
Pre	0.0742 (0.0933)	0.0532 (0.0567)	0.0899 (0.1252)
Post	0.0669 (0.0711)	0.1109 (0.1463)	0.0834 (0.0730)
Change	−0.0072 (0.0723)	0.0561 (0.1600)	−0.0117 (0.1093)
Form Biofilms			
Pre	0.1735 (0.0900)	0.1665 (0.1184)	0.2947 (0.1391)
Post	0.1495 (0.0739)	0.1914 (0.1481)	0.2464 (0.1036) *
Change	−0.0240 (0.0881)	0.0203 (0.1562)	−0.0584 (0.1171)
Gram Negative			
Pre	0.6103 (0.1102)	0.5516 (0.1705)	0.6545 (0.0707)
Post	0.5561 (0.0872)	0.5684 (0.1867)	0.6305 (0.1098)
Change	−0.0542 (0.1167)	−0.0012 (0.2010)	−0.0224 (0.08892)
Gram Positive			
Pre	0.3896 (0.1102)	0.4483 (0.1705)	0.3454 (0.0707)
Post	0.4438 (0.0872)	0.4315 (0.1867)	0.3694 (0.1098)
Change	0.0542 (0.1167)	0.0012 (0.2010)	0.0224 (0.0889)
Potentially Pathogenic			
Pre	0.6429 (0.1171)	0.5674 (0.1499)	0.6053 (0.1164)
Post	0.5874 (0.1167) *	0.6116 (0.1621)	0.6145 (0.0887)
Change	−0.0555 (0.1280)	0.0305 (0.1768)	0.0142 (0.1466)
Stress Tolerant			
Pre	0.1063 (0.0863)	0.0947 (0.0557)	0.1726 (0.1306)
Post	0.0695 (0.0594) *	0.1360 (0.1535)	0.1182 (0.0859)
Change	−0.0368 (0.0575) ^ab^	0.0376 (0.1501) ^b^	−0.0574 (0.1232) ^a^

Differences between the groups were analyzed by a one-way ANOVA followed by a Duncan’s post hoc test. Values with different uppercase letters (^a,b^ indicate statistically significant difference between the groups (*p* < 0.05) at Duncan’s test. Wilcoxon signed-rank test was used to calculate differences between baseline and the end of the intervention: * *p* < 0.05.

## Data Availability

Raw 16S rRNA sequencing data used in this study are openly available in the NCBI short read archive under ID study: PRJNA634244.

## Supplementary Materials

The following are available online at https://www.mdpi.com/2075-4426/11/2/109/s1, Figure S1: (**a**) Relative abundance of the members of core microbiome after each intervention at baseline and the end of the study at family level. MedDiet: Mediterranean Diet group (*n* = 21). BS: Bariatric surgery group (*n* = 22). VLCKD: very-low calorie ketogenic diet group (*n* = 18). (**b**) Relative abundance of the members of core microbiome after each intervention at baseline and the end of the study at genus level. MedDiet: Mediterranean Diet group (*n* = 21). BS: Bariatric surgery group (*n* = 22). VLCKD: very-low calorie ketogenic diet group (*n* = 18). (**c**) Relative abundance of the members of core microbiome after each intervention at baseline and the end of the study at specie level. MedDiet: Mediterranean Diet group (*n* = 21). BS: Bariatric surgery group (*n* = 22). VLCKD: very-low calorie ketogenic diet group (*n* = 18). Table S1: Description of the pathways altered in each intervention.

## Abbreviations

BMI	body mass index
BS	bariatric surgery
CRP	C-reactive protein
KEGG	Kyoto Encyclopedia of Genes and Genomes
HDL-cholesterol	High density lipoprotein cholesterol
LDL-cholesterol	Low density lipoprotein cholesterol
MedDiet	hypocaloric Mediterranean diet
VLCKD	very-low-calorie ketogenic diet (VLCKD)
