# Supplementary material for: Different Weight Loss Intervention Approaches Reveal a Lack of a Common Pattern of Gut Microbiota Changes
Source: J Pers Med. 2021 Feb 8;11(2):109. doi: 10.3390/jpm11020109 (PMC7915884; doi:10.3390/jpm11020109)
Supplement: Supplementary file 1 [file jpm-11-00109-s001.pdf]

Supplementary Table S1: Description of the pathways altered in each intervention

COMMON NAME	Pathway ID	MedDiet	Superclasses
L-lysine biosynthesis I	DAPLYSINESYN-PWY	↑	Biosynthesis → Amino Acid Biosynthesis → Proteinogenic Amino Acid Biosynthesis → L-lysine Biosynthesis
Superpathway of glycol metabolism and degradation	GLYCOL-GLYOXDE-PWY	↓	Degradation/Utilization/Assimilation → Alcohol Degradation
Heterolactic fermentation	P122-PWY	↑	Generation of Precursor Metabolite and Energy → Fermentation → Fermentation of Pyruvate → Pyruvate Fermentation to Ethanol<br>Generation of Precursor Metabolite and Energy → Fermentation → Fermentation to Alcohols → Pyruvate Fermentation to Ethanol<br>Generation of Precursor Metabolite and Energy → Fermentation → Fermentation to Short-Chain Fatty Acids → Fermentation to Lactate
Bifidobacterium shunt	P124-PWY	↑	Degradation/Utilization/Assimilation → Carbohydrate Degradation → Sugar Degradation<br>Degradation/Utilization/Assimilation → Carboxylate Degradation → Fermentation to Acetate<br>Generation of Precursor Metabolite and Energy → Fermentation → Fermentation to Short-Chain Fatty Acids → Fermentation to Acetate<br>Generation of Precursor Metabolite and Energy → Fermentation → Fermentation to Short-Chain Fatty Acids → Fermentation to Lactate
Superpathway of phospholipid biosynthesis I (bacteria)	PHOSLIPSYN-PWY	↑	Biosynthesis → Fatty Acid and Lipid Biosynthesis → Phospholipid Biosynthesis
Reductive TCA cycle II	PWY-5392	↑	Degradation/Utilization/Assimilation → C1 Compound Utilization and Assimilation → CO2 Fixation → Autotrophic CO2 Fixation → Reductive TCA Cycles
Acetyl-CoA fermentation to butanoate II	PWY-5676	↑	Generation of Precursor Metabolite and Energy → Fermentation → Fermentation to Short-Chain Fatty Acids → Fermentation to Butanoate
Succinate fermentation to butanoate	PWY-5677	↑	Generation of Precursor Metabolite and Energy → Fermentation → Fermentation to Short-Chain Fatty Acids → Fermentation to Butanoate
Superpathway of 2,3-butanediol biosynthesis	PWY-6396	↑	Biosynthesis → Other Biosynthesis → Butanediol Biosynthesis
Superpathway of purine deoxyribonucleosides degradation	PWYO-1297	↑	Degradation/Utilization/Assimilation → Nucleoside and Nucleotide Degradation
Peptidoglycan maturation (meso-diaminopimelate containing)	PWYO-1586	↑	Biosynthesis → Cell Structure Biosynthesis → Cell Wall Biosynthesis → Peptidoglycan Biosynthesis
Phosphatidylglycerol biosynthesis I (plastidic)	PWY4FS-7	↑	Biosynthesis → Fatty Acid and Lipid Biosynthesis → Phospholipid Biosynthesis → Phosphatidylglycerol Biosynthesis
Phosphatidylglycerol biosynthesis II (non-plastidic)	PWY4FS-8	↑	Biosynthesis → Fatty Acid and Lipid Biosynthesis → Phospholipid Biosynthesis → Phosphatidylglycerol Biosynthesis

COMMON NAME	Pathway ID	BS	Superclasses
O-antigen building blocks biosynthesis (E. coli)	OANTIGEN-PWY	↓	Biosynthesis → Carbohydrate Biosynthesis → Sugar Biosynthesis → Sugar Nucleotide Biosynthesis<br><br>Metabolic Clusters
Glycolysis V (Pyrococcus)	P341-PWY	↓	Generation of Precursor Metabolite and Energy → Glycolysis
Pentose phosphate pathway	PENTOSE-P-PWY	↑	Generation of Precursor Metabolite and Energy → Pentose Phosphate Pathways
Superpathway of CDP-glucose-derived O-antigen building blocks biosynthesis	PWY-5823	↑	Biosynthesis → Carbohydrate Biosynthesis → Sugar Biosynthesis → Sugar Nucleotide Biosynthesis → CDP-sugar Biosynthesis
Archaetidylinositol biosynthesis	PWY-6350	↓	Biosynthesis → Fatty Acid and Lipid Biosynthesis → Phospholipid Biosynthesis
Mono-trans, poly-cis decaprenyl phosphate biosynthesis	PWY-6383	↓	Biosynthesis → Cofactor, Prosthetic Group, Electron Carrier, and Vitamin Biosynthesis → Polyprenyl Biosynthesis
Phosphopantothenate biosynthesis III (archaebacteria)	PWY6654	↓	Biosynthesis → Cofactor, Prosthetic Group, Electron Carrier, and Vitamin Biosynthesis → Vitamin Biosynthesis → Phosphopantothenate Biosynthesis
L-rhamnose degradation II	PWY-6713	↓	Degradation/Utilization/Assimilation → Carbohydrate Degradation → Sugar Degradation → L-rhamnose Degradation
7-(3-amino-3-carboxypropyl)-wyosine biosynthesis	PWY-7286		Macromolecule Modification → Nucleic Acid Processing
Taxadiene biosynthesis (engineered)	PWY-7392	↓	Biosynthesis → Secondary Metabolite Biosynthesis → Terpenoid Biosynthesis → Diterpenoid Biosynthesis
L-methionine salvage cycle I (bacteria and plants)	PWY-7528	↑	Biosynthesis → Amino Acid Biosynthesis → Proteinogenic Amino Acid Biosynthesis → L-methionine Biosynthesis → L-methionine Salvage
UDP-N-acetyl-D-glucosamine biosynthesis I	UDPNAGSYN-PWY	↓	Biosynthesis → Carbohydrate Biosynthesis → Sugar Biosynthesis → Sugar Nucleotide Biosynthesis → UDP-sugar Biosynthesis → UDP-N-acetyl-D-glucosamine Biosynthesis

COMMON NAME	Pathway ID	VLCKD	Superclasses
Glycolysis III (from glucose)	ANAGLYCOLYSIS -PWY	↑	Generation of Precursor Metabolite and Energy → Glycolysis
L-methionine biosynthesis I	HOMOSER-METSYN-PWY	↓	Biosynthesis → Amino Acid Biosynthesis → Proteinogenic Amino Acid Biosynthesis → L-methionine Biosynthesis → L-methionine De Novo Biosynthesis
Superpathway of S-adenosyl-L-methionine biosynthesis	MET-SAM-PWY	↓	Superpathways
Entner-Doudoroff pathway II (non-phosphorylative)	NPGLUCAT-PWY	↓	Degradation/Utilization/Assimilation → Carbohydrate Degradation → Sugar Degradation → Entner-Duodoroff Pathways<br>Generation of Precursor Metabolite and Energy → Entner-Duodoroff Pathways
Superpathway of (R,R)-butanediol biosynthesis	P125-PWY	↑	Biosynthesis → Other Biosynthesis → Butanediol Biosynthesis
superpathway of N-Acetylneuraminate degradation	P441-PWY	↑	Degradation/Utilization/Assimilation → Carboxylate Degradation
pentose phosphate pathway	PENTOSE-P-PWY	↑	Generation of Precursor Metabolite and Energy → Pentose Phosphate Pathways
Urea cycle	PWY-4984	↑	Degradation/Utilization/Assimilation → Inorganic Nutrient Metabolism → Nitrogen Compound Metabolism
S-adenosyl-L-methionine cycle I	PWY-6151	↓	Biosynthesis → Amino Acid Biosynthesis → Proteinogenic Amino Acid Biosynthesis → L-methionine Biosynthesis → L-methionine Salvage → S-adenosyl-L-methionine cycle
Starch biosynthesis	PWY-622	↑	Biosynthesis → Carbohydrate Biosynthesis → Glycan Biosynthesis → Polysaccharide Biosynthesis → Glycogen and Starch Biosynthesis<br>Biosynthesis → Carbohydrate Biosynthesis → Polysaccharide Biosynthesis → Glycogen and Starch Biosynthesis<br>Glycan Pathways → Glycan Biosynthesis → Polysaccharide Biosynthesis → Glycogen and Starch Biosynthesis
CMP-legionaminate biosynthesis I	PWY-6749	↑	Biosynthesis → Carbohydrate Biosynthesis → Sugar Biosynthesis → Sugar Nucleotide Biosynthesis → CMP-sugar Biosynthesis → CMP-legionaminate biosynthesis
Superpathway of glucose and xylose degradation	PWY-6901	↑	Degradation/Utilization/Assimilation → Carbohydrate Degradation → Sugar Degradation
paromamine biosynthesis II	PWY-7022	↑	Biosynthesis → Secondary Metabolite Biosynthesis → Antibiotic Biosynthesis → Paromamine Biosynthesis
UDP-2,3-diacetamido-2,3-dideoxy-α-D-mannuronate biosynthesis	PWY-7090	↑	Biosynthesis → Carbohydrate Biosynthesis → Sugar Biosynthesis → Sugar Nucleotide Biosynthesis → UDP-sugar Biosynthesis
Superpathway of pyrimidine deoxyribonucleotides de novo biosynthesis	PWY-7211	↓	Biosynthesis → Nucleoside and Nucleotide Biosynthesis → 2'-Deoxyribonucleotide Biosynthesis → Pyrimidine Deoxyribonucleotide De Novo Biosynthesis

			Biosynthesis → Nucleoside and Nucleotide Biosynthesis → Pyrimidine Nucleotide Biosynthesis → Pyrimidine Nucleotide De Novo Biosynthesis → Pyrimidine Deoxyribonucleotide De Novo Biosynthesis
Superpathway of UDP-N-acetylglucosamine-derived O-Antigen building blocks biosynthesis	PWY-7332	↑	Biosynthesis → Carbohydrate Biosynthesis → Sugar Biosynthesis → Sugar Nucleotide Biosynthesis → UDP-sugar Biosynthesis
β-(1,4)-mannan degradation	PWY-7456	↑	Degradation/Utilization/Assimilation → Carbohydrate Degradation → Polysaccharide Degradation<br>Degradation/Utilization/Assimilation → Polymeric Compound Degradation → Polysaccharide Degradation
tRNA processing	PWYO-1479	↓	Macromolecule Modification → Nucleic Acid Processing
